# Surveillance and diagnosis of zoonotic foodborne parasites

**DOI:** 10.1002/fsn3.530

**Published:** 2017-11-12

**Authors:** Reza Zolfaghari Emameh, Sami Purmonen, Antti Sukura, Seppo Parkkila

**Affiliations:** ^1^ Department of Energy and Environmental Biotechnology Division of Industrial & Environmental Biotechnology National Institute of Genetic Engineering and Biotechnology (NIGEB) Tehran Iran; ^2^ Faculty of Medicine and Life Sciences University of Tampere Tampere Finland; ^3^ Department of Veterinary Biosciences Faculty of Veterinary Medicine University of Helsinki Helsinki Finland; ^4^ Fimlab Laboratories Ltd and Tampere University Hospital Tampere Finland

**Keywords:** diagnosis, fish, food, PCR (polymerase chain reaction), quality control

## Abstract

Foodborne parasites are a source of human parasitic infection. Zoonotic infections of humans arise from a variety of domestic and wild animals, including sheep, goats, cattle, camels, horses, pigs, boars, bears, felines, canids, amphibians, reptiles, poultry, and aquatic animals such as fishes and shrimp. Therefore, the implementation of efficient, accessible, and controllable inspection policies for livestock, fisheries, slaughterhouses, and meat processing and packaging companies is highly recommended. In addition, more attention should be paid to the education of auditors from the quality control (QC) and assurance sectors, livestock breeders, the fishery sector, and meat inspection veterinarians in developing countries with high incidence of zoonotic parasitic infections. Furthermore, both the diagnosis of zoonotic parasitic infections by inexpensive, accessible, and reliable identification methods and the organization of effective control systems with sufficient supervision of product quality are other areas to which more attention should be paid. In this review, we present some examples of successful inspection policies and recent updates on present conventional, serologic, and molecular diagnostic methods for zoonotic foodborne parasites from both human infection and animal‐derived foods.

## 1 INTRODUCTION

Zoonotic parasites are among the most important causes of human infectious disease, especially in poor and developing countries (Jenkins et al., 2013). Since meat was added to the human diet, it caused the emergence of zoonotic foodborne parasites from animal‐derived foods among the human population and consequently, much research worldwide has been focused on zoonotic foodborne parasites. The World Health Organization (WHO) reported some important meat‐ and fish‐borne parasites in 2002, including *Anisakis* spp., *Capillaria philippinensis*,* Clonorchis sinensis*,* Diphyllobothrium* spp., *Echinococcus* spp., *Fasciola* spp., *Fasciolopsis buski*,* Gnathostoma* spp., *Opisthorchis* spp., *Sarcocystis* spp., *Spirometra* spp., *Taenia* spp., *Toxoplasma gondii*,* Trichinella* spp., and blood flukes (Dorny, Praet, Deckers, & Gabriel, 2009). Several other soil‐transmitted and waterborne food parasites have been reported and added to the previous list from the WHO, such as *Ascaris lumbricoides, Cryptosporidium* spp.*, Entamoeba* spp., *Giardia* spp.*, Hymenolepis nana, Paragonimus* spp., pentastomids (tongue worms), *Strongyloides stercoralis,* and *Toxocara* spp. (Jenkins et al., 2013; Murrell, 2013; Sithithaworn et al., 2006). In their last update in July 2014, the Food and Agriculture Organization (FAO) and the WHO reported a “top ten” list of foodborne parasites of greatest global concern, which affect the health of millions of people by infecting tissues and causing clinical symptoms (FAO, 2014). The rankings of the “top ten” foodborne parasites by the FAO‐WHO was based on the parasite burden in humans. The “top ten” foodborne parasites with same examples from which sources man can be infected are as follows:

1. *Taenia solium* (pork tapeworm): In pork
2. *Echinococcus granulosus* (hydatid worm or dog tapeworm): vegetables, fruits, and other food infected with the eggs of the hydatid worm or handling dogs
3. *Echinococcus multilocularis* (a type of tapeworm): the same as *E. granulosus*
4. *Toxoplasma gondii* (protozoa): In the meat of small ruminants, pork, beef, and their organs, as well as unpasteurized milk and cheese from goats or contaminated vegetables and other food, soil, etc.
5. *Cryptosporidium* spp.(protozoa): In uncooked meat products, water, fruit juice, and milk
6. *Entamoeba histolytica* (protozoa): In uncooked infected meat products, water, and sexual transmission
7. *Trichinella* spp. (pork worm): In pork, dogs, foxes, horses, wild boars, walruses, and birds
8. Opisthorchiidae (Trematoda, family of flatworms): In freshwater fishes
9. *Ascaris lumbricoides* (small intestinal roundworms): contaminated food, water, vegetables, environment
10. *Trypanosoma cruzi* (protozoa, Chagas diseases): mainly insect vector‐borne disease, but may be also from contaminated food and drinks

These zoonotic foodborne parasites potentially represent a critical danger for human health if infected tissue is consumed undercooked or raw and threaten the safety of animal‐derived foods (Dorny et al., 2009; Jenkins et al., 2013; Murrell, 2013; Sithithaworn et al., 2006). Therefore, the control of foodborne parasites is a high priority for quality assessment in slaughterhouses and food companies. On the other hand, governments incur less cost from the diagnosis and prevention of parasite‐infected animal‐derived foods than from the treatment of parasitic infections. The simplest of the different types of preventive actions for the detection of the zoonotic parasites from animal‐derived foods is a direct visual inspection of an animal carcass. Although visual inspection gives a good holistic evaluation of carcass quality and robust safety information for specific parasites, it has disadvantages such as impossibility in detection of some of the parasites infecting stages. It is labor intensive, especially when direct microscopy is necessary, and also has sensitivity and specificity limitations (Dorny et al., 2009). The development of molecular biology techniques and medical engineering during the last decades allow the diagnosis of foodborne parasites to be conducted by cheap, less labor intensive, and reliable molecular‐based techniques for the infected samples with animal or human origin, but not for foods.

Some of the “top ten” parasites such as *Echinococcus* spp., *Cryptosporidium* spp., *E. histolytica*,* A. lumbricoides*, and *T. cruzi* are mainly waterborne or soil borne parasites. In this review, our main focus is on the major zoonotic foodborne parasites. We evaluate the present inspection status of animal‐derived foods and identify conventional and advanced diagnostic methods to detect zoonotic foodborne parasites from both human infections and animal‐derived foods.

### 1.1 Monitoring and inspection

The major reason for the monitoring and inspection of animal‐derived foods is to guarantee food safety (Berends & Van Knapen, 1999; Naugle, Holt, Levine, & Eckel, 2005). As long as man has eaten meat, some kind of evaluation of safety and meat quality has most likely been carried out. Early control systems are in religious texts, which gives rules for the edibility of meat (Eliasi & Dwyer, 2002; Farouk, 2013). In this century in Europe, the European Commission has implemented a regulatory program 178/2002 to establish the general principles and requirements of food laws (Frentzel, Menrath, Tomuzia, Braeunig, & Appel, 2013), and all of the EU member states have had their own meat inspection systems long before the European common system. For example, in Finland, trichinella inspection started in 1867, and in addition to imported meat, domestic animals are controlled currently for presence of trichinella infection (Sukura, Nareaho, Mikkonen, Niemi, & Oivanen, 2002). The scientific panels and committees of European Food Safety Authority (EFSA) also advised the procedures for matters of food safety to European member states. The meat regulatory and inspection programs (regulation numbers 853/2004 and 854/2004) were approved by the European parliament in 2004. These programs were established to officially determine the requirements for the hygienic control of consumable products of animal origin for human consumption. They encompass all animal‐derived foods, including minced meat, mechanically separated meat, live bivalve mollusks, fishery products, frogs’ legs, snails, rendered animal fats and greaves, treated stomachs, bladders, intestines, gelatin, and collagen. The inspection programs have been written based on the HACCP (Hazard Analysis and Critical Control Point) system to evaluate all aspects of the physical hazards, patho‐physiological abnormalities, and chemo‐microbiological contamination of meat during slaughtering, cutting and boning, marking, as well as storage, transport and maturation, to prevent the introduction of unsanitary meat in the human food chain. These inspection rules were included in a package of measures by the European Commission in 2013. As official inspectors, veterinarians must pass a qualification exam organized by a competent authority to perform a valid inspection. The exam encompasses community legislation on health, good hygiene and farming practices, HACCP principles, epidemiology, and transmissible spongiform encephalitis (TSEs). Moreover, veterinarian inspectors should have at least 200 hours of practical training before they are allowed to work in the field. Fishery, poultry, and livestock products should be declared free from any parasitic infection to ensure that these products are non‐hazardous for human consumption.

Many national food companies and surveillance‐based systems for monitoring and controlling the quality of slaughter processes and produced meat exist. Of all the numerous, present worldwide inspection methods, four have been selected and described below:

1. *Nergal‐Abattoir* is a compulsory regulatory system that was designed by the French Ministry of Agriculture and implemented in several slaughterhouses (Dupuy et al., 2013). It has been a potent meat surveillance and inspection project in France since 2006. This online system collects demographic data such as age, sex, and breed and health related data of slaughtered cattle.
2. Advanced technologies have been developed during the past decade for the detection of hidden zoonotic foodborne parasites in animal‐derived foods during safety inspections (Elmasry, Barbin, Sun, & Allen, 2012; Feng & Sun, 2012) such as near‐infrared hyperspectral imaging for the rapid assessment of pork meat quality (Barbin, Elmasry, Sun, & Allen, 2012; Huang, Liu, & Ngadi, 2014).
3. In 1996, PulseNet USA was established by US Department of Agriculture (USDA) and the Center for Disease Control and Prevention (CDC) to control food safety (Ransom & Kaplan, 1998), along with veterinary inspection and laboratory diagnostic techniques. Pulsed‐field gel electrophoresis (PFGE) (a molecular subtyping method) has been used for the detection of foodborne bacterial infections, such as *Escherichia coli* O157:[H7] (STEC O157), *Salmonella enterica*,* Listeria monocytogenes*,* Shigella* spp., and *Campylobacter* spp. (Gerner‐Smidt et al., 2006). Presently, 50 US state and public health laboratories (PHLs) and those in many other countries worldwide have been connected by the PulseNet surveillance network for foodborne infections to share all genome sequencing information (Boxrud, Monson, Stiles, & Besser, 2010), which could provide a unique means to control zoonotic foodborne parasitic infection as well.
4. Although, there is limited evidence on effective auditory systems in the slaughterhouses and meat markets of low‐income countries, projects have been performed by the International Livestock Research Institute (ILRI) in Africa and Asia to define the hazardous foodborne parasites, which can be transmitted through animal‐derived foods, such as meat, fish, and crustaceans. In those countries, foodborne parasites can be transmitted from infected people or animals of wet markets, where many people do not comply governmental regulations, and consequently escape from effective health and safety auditory systems. It seems that improving the existing auditory systems may be more successful than attempting to implement completely new regulations (Grace, 2015).

### 1.2 Diagnosis of zoonotic foodborne parasites from both human infection and animal‐derived foods

**Figure 1:**
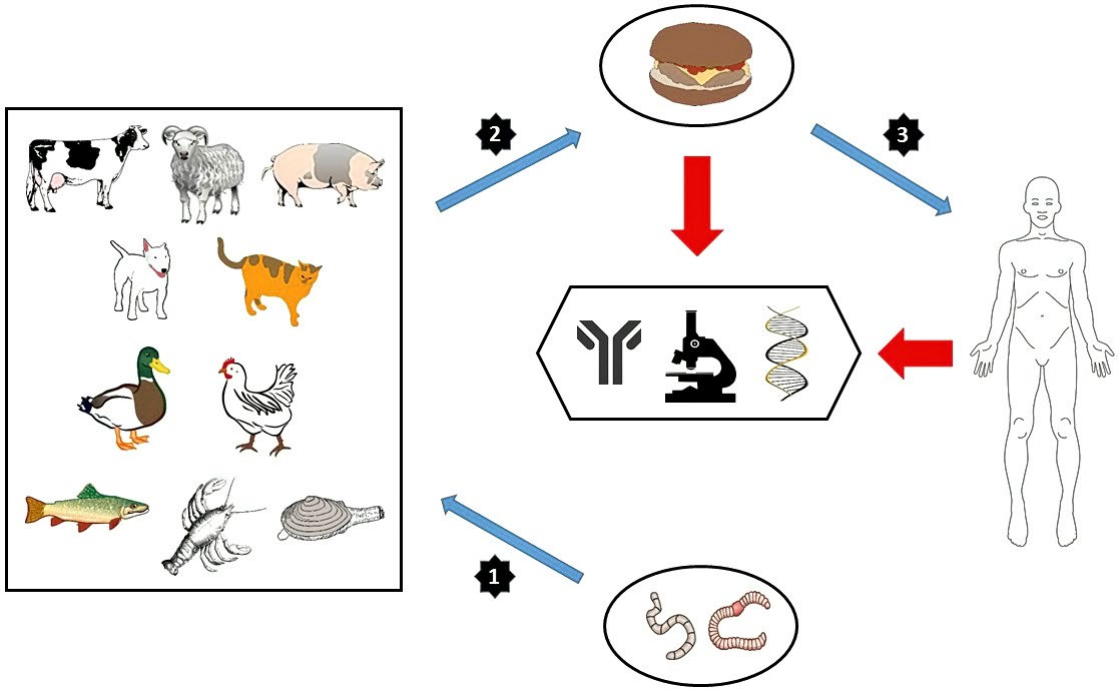
Schematic diagram of transmission and diagnosis of zoonotic foodborne parasites. Blue arrows show: (1) Transmission of parasites to animals, (2) transmission of parasites to animal‐derived foods, and (3) transmission of parasites from animal‐derived foods to human. Red arrow shows the possibility of diagnosis of zoonotic foodborne parasites by direct microscopic, serologic, and molecular‐based methods

The control of animal‐derived foods for the presence of zoonotic foodborne parasites is a high‐priority for the safety and quality assessment in slaughterhouses and food companies. The development of molecular biology techniques in the last decades has led to the goal that the diagnosis of zoonotic foodborne parasites in animal‐derived foods be conducted by cheap and reliable molecular‐based methods instead of the conventional, time‐consuming methods. For example, based on European Community Regulation No. 2075/2005, DNA analysis is used for the identification of *Trichinella* larvae at the species level from muscle samples (Pozio, Rossi, & Dipartimento Di Malattie Infettive Parassitarie E Immunomediate, 2008). Some laboratory techniques that are used to diagnose human infections can also be applied for the detection of parasites in food animals. To design new molecular‐based diagnostic methods to detect foodborne parasites, we should understand the present status of diagnostic assays for each parasite. In this review, we classified the zoonotic foodborne parasites from both human infections and animal‐derived foods as helminths (Trematoda, Cestoda, and Nematoda) and protozoans. Additionally, we presented the latest progress in conventional, serologic, and molecular diagnostic methods for detection of parasites from both human infection and animal‐derived foods (Figure 1). A summary of these diagnostic methods is shown in Table 1.

**Table 1:** The diagnostic methods for the detection of the selected zoonotic foodborne parasites from both infections and/or animal‐derived foods

Parasite	Classification	Conventional methods	Serologic methods	Molecular methods
*Clonorchis sinensis*	Trematoda	Gastroscopy and biopsy (Nie et al., 2014)	ELISA (Nie et al., 2014)	Real‐time PCR (Kim et al., 2009) HRM (Cai et al., 2014) LAMP (Cai et al., 2010) NGS (Kim et al., 2014)
*Opisthorchis viverrini*	Trematoda	Microscopic assay (Worasith et al., 2015) FECT (Worasith et al., 2015)	ELISA (Chaiyarit et al., 2011; Worasith et al., 2015) OV‐ES assay (Worasith et al., 2015)	PCR (Parvathi et al., 2008) LAMP (Arimatsu et al., 2012, 2015) NGS (Young et al., 2010)
*Paragonimus* spp.	Trematoda	Microscopic assay (Slesak et al., 2011)	Western blot (Fischer et al., 2013)	PCR (Intapan et al., 2012) NGS (McNulty et al., 2014)
*Diphyllobothrium* spp.	Cestoda	Microscopic assay (Waki et al., 1986) Medical imaging (Waki et al., 1986) Blood Vit B12 level (Jimenez, Rodriguez, Gamboa, Rodriguez, & Garcia, 2012)	NA^a^	PCR (Chen et al., 2014; Guo et al., 2012)
*Taenia* spp.	Cestoda	Microscopic assay (Mayta et al., 2008)	ELISA (Abuseir et al., 2007; Mayta et al., 2008) EITB (Deckers et al., 2009; Liu et al., 2017; Pathak et al., 1994)	PCR (Abuseir et al., 2006; Chiesa et al., 2010) PCR‐RFLP (Rodriguez‐Hidalgo et al., 2002) Nested PCR (Mayta et al., 2008)
*Anisakis simplex*	Nematoda	Gastroscopy (Armentia et al., 2006; Gamboa et al., 2012) Histopathology (Armentia et al., 2006; Gamboa et al., 2012)	RIA (Armentia et al., 2006; Gamboa et al., 2012) ELISA (Cuellar et al., 2012)	NA^a^
*Gnathostoma* spp.	Nematoda	Eosinophil count (Graeff‐Teixeira et al., 2009; Kuberski, 1979)	Western blot (Graeff‐Teixeira et al., 2009) Immunoblot (Intapan et al., 2010; Janwan et al., 2013) ELISA (Graeff‐Teixeira et al., 2009) Two‐dimensional PAGE (Graeff‐Teixeira et al., 2009)	NA^a^
*Trichinella* spp.^b^	Nematoda	Microscopic assay (Nockler, Pozio, Voigt, & Heidrich, 2000)	ELISA (Gamble et al., 2000; Korinkova et al., 2008; Van De et al., 2012) IFA (Zhang et al., 2013)	PCR (Li et al., 2010; Lin et al., 2013; Tantrawatpan et al., 2012; Zolfaghari Emameh et al., 2015) RT‐PCR (Zhang et al., 2013) Real‐time PCR (Guenther et al., 2008; Lin et al., 2013; Tantrawatpan et al., 2012) LAMP (Lin et al., 2013; Tantrawatpan et al., 2012) Multiplex PCR (Blaga et al., 2009; Borsuk et al., 2003; Zarlenga et al., 1999) RLB (Rombout et al., 2001)
*Sarcocystis* spp.^c^	Protozoa	Microscopic assay (Bunyaratvej et al., 2007; Hamidinejat et al., 2013; Nourollahi Fard et al., 2009)	ELISA (Habeeb et al., 1996; Tenter, 1987) IFAT (Habeeb et al., 1996; Tenter, 1987)	PCR (More et al., 2014) Real‐time PCR (More et al., 2014) RT‐PCR (Vangeel et al., 2007)
*Toxoplasma gondii* d	Protozoa	Microscopic assay (Van De Ven et al., 1991)	Sabin‐Feldman dye test (Udonsom et al., 2010) ELISA (Chen et al., 2008; Wang et al., 2011; Warnekulasuriya et al., 1998) IFA (Bayarri et al., 2012) ICS (Wang et al., 2011)	MC‐PCR (Aroussi et al., 2015) Real‐time PCR (Jauregui et al., 2001) NGS (Cheng et al., 2015)

a Not defined.

b Proteomic methods have also been considered as other diagnostic methods for the detection of trichinellosis (Wang et al., 2014).

c A new approach and diagnosis method to detect toxin in infected tissues showed a 15‐kDa protein from *S. fayeri* cysts, which was homologous to the actin‐depolymerizing factor of *T. gondii* and *Eimeria tenella* (Kamata et al., 2014).

d A *T. gondii* infection can be identified by other methods such as inoculation in mice, tissue culture, and a hybridization assay (Van De Ven et al., 1991).

## 2 HELMINTHS

### 2.1 Trematoda

Approximately a hundred species of foodborne trematodes infect humans (Fried & Abruzzi, 2010; Keiser, Duthaler, & Utzinger, 2010), and some examples are discussed herein.

#### 2.1.1 *Clonorchis sinensis*

*Clonorchis sinensis* (also known as the Chinese or oriental liver fluke) is a liver fluke that causes clonorchiasis (Tang, Huang, & Yu, 2016). The life cycle of this fish‐borne trematode includes the egg (produced by the adult helminths in definitive hosts in an aquatic environment); the miracidium, sporocyst, redia, and cercaria (these four stages occur in freshwater snails); and the metacercaria (in freshwater fish and occasionally in shrimp). Humans can be infected after eating undercooked freshwater fish or shrimp. The adult helminths are typically localized in the human liver, bile duct, and gall bladder (Hong & Fang, 2012). The characteristic histopathological symptoms of infection include bile duct epithelial proliferation, which is followed by periductal fibrosis (Choi, Han, Hong, & Lee, 2004). Additionally, edema is frequently present in the biliary epithelium. Therefore, histopathological changes are the major clinical features of clonorchiasis. This parasite is considered as a causative agent of liver and bile duct cancer (cholangiocarcinoma), and the International Agency for Research on Cancer has classified *C. sinensis* as a group 1 biological carcinogen (Bouvard et al., 2009). The main diagnostic method for a *C. sinensis* infection is the identification of parasite eggs in fecal, bile, and duodenal biopsy samples, which is considered the gold standard for the diagnosis of human clonorchiasis (Nie et al., 2014). An ELISA‐based detection of IgY (egg yolk immunoglobulin) against the cysteine proteinase of *C. sinensis* detects a circulating antigen in serum samples of patients with clonorchiasis. The results from this IgY‐based immunomagnetic bead ELISA system (IgY‐IMB‐ELISA) showed a sensitivity of 93.3% in cases of severe infection (5,000 to 9,999 eggs per gram of feces (epg)), 86.7% in cases of moderate infection (1,000–4,999 epg), and 75.0% in cases of mild infection (<1,000 epg) of clonorchiasis. As another attempt to design a new sensitive method for the diagnosis of clonorchiasis, a real‐time PCR method has been used to detect the internal transcribed spacer 2 sequence (ITS2) from stool samples infected with *C. sinensis* (Kim et al., 2009). The sensitivity of the assay was 100%, and three out of the 26 samples that appeared egg‐negative by direct microscopic method were found to be PCR‐positive. In another study, a real‐time PCR and high resolution melting (HRM) analysis were used for the specific detection and rapid identification of *C. sinensis* and *Opisthorchis viverrini* (Cai et al., 2014). Primers targeting the cytochrome c oxidase subunit 1 (*cox1*) gene were highly specific for these liver flukes, with no amplification of closely related trematodes. This method has a detection limit below 1 pg of genomic DNA or 5 eggs per gram of stool, or 1 metacercaria of *C. sinensis*. In addition, *C. sinensis* and *O. viverrini* were differentiated by their HRM pattern. A loop‐mediated isothermal amplification (LAMP) was used for rapid and sensitive detection from the source of infection in the fish (Cai et al., 2010). The *cox1* gene was targeted for diagnosis in this method. The sensitivity of the method was determined as 10^−8^ ng/μl for detection of genomic DNA of *C. sinensis* metacercariae. The robust results of the method showed that LAMP has the potential to be applied for the detection of human clonorchiasis as well. Next‐generation sequencing (NGS) techniques have revolutionized molecular‐based detection of parasitic infections including clonorchiasis. ClonorESTdb database has been established as the collection of *C. sinensis* expressed sequence tags (ESTs) for genomics studies (Kim et al., 2014; Young et al., 2010). These novel techniques represent robust and cost effective choices for detection of ESTs of *C. sinensis* in biological specimens.

At the moment, only a human *C. sinensis* antibody (IgG) ELISA kit is commercially available in the market for diagnosis or research purposes.

#### 2.1.2 *Opisthorchis viverrini*

*Opisthorchis viverrini* is a liver fluke and a fish‐borne trematode that is the causative agent of human opisthorchiasis. The life cycle of this trematode is similar to *C. sinensis*, in which a freshwater fish acts as an intermediate and humans as the ultimate host (Sripa et al., 2007). Humans are infected through the ingestion of raw or insufficiently cooked fish containing trematode larvae (metacercariae) (Worasith et al., 2015). *O. viverrini* is the etiological agent of cholangiocarcinoma (a liver cancer subtype) (Sripa et al., 2007). The human immune response mediates some of the hepatobiliary features of opisthorchiasis such as chronic inflammation around the biliary tree and severe hyperplasia of the cholangiocytes (Glaser, Gaudio, Miller, Alvaro, & Alpini, 2009).

The routine detection method for larvae in fish is the enzymatic digestion of infected fish tissue, sedimentation, and direct microscopy techniques (Sripa et al., 2007). In human infections, the present gold standard for the detection of *O. viverrini* is the formalin ethyl‐acetate concentration (FECT) method for stool samples and the identification of parasite eggs by a direct microscopic method (Worasith et al., 2015).

ELISA has been used for measurement of *O. viverrini* excretory‐secretory antigens (OV‐ES Ags) in urine (urine OV‐ES assay) in the serologic identification of opisthorchiasis. The OV‐ES assay had a higher sensitivity (81%) and specificity (70%) than the FECT gold standard for the diagnosis of opisthorchiasis in urine (Worasith et al., 2015). It has been shown that the antibodies (IgA and IgG) against *O. viverrini* are present in patient's saliva as well as serum (Chaiyarit, Sithithaworn, Thuwajit, & Yongvanit, 2011). Similar approach with some modifications may be utilized for serodiagnostics of *O. viverrini* in meat extracts.

For a molecular diagnosis of this infection, genomic DNA was isolated from adult and larval trematodes. Genomic regions were randomly selected for a PCR‐based diagnosis method. The assay was Opisthorchis‐specific with no false‐positive cross‐reaction with other fish‐infecting trematodes including *C. sinensis*. The sensitivity of the method was 10^−12^ ng of parasite DNA. Another detection technique that has been used is LAMP, a colorimetric‐based method (Arimatsu, Kaewkes, Laha, & Sripa, 2015). In this method, genomic DNA was isolated from an adult trematode. Microsatellite 2 (OVMS2) and microsatellite 6 (OVMS6) were selected as the targets for designing the LAMP primers. The respective specificity and sensitivity of the test was 61.5% and 100%. The sensitivity was clearly higher than that of the microscopic egg examination. Using the hydroxyl naphthol blue (HNB)‐LAMP, microsatellite 6 (OVMS6) could specifically amplify a target site from the *O. viverrini* genome, and no false‐positive results have been identified from other parasites, such as *O. felineus, C. sinensis, Centrocestus caninus, Fasciola gigantica, Haplorchis taichui,* and *Haplorchoodes* spp. In addition, the LAMP technique has been evaluated for stool samples, and the sensitivity of the assay was 100%. Additionally, 38.5% of microscopically negative samples were LAMP‐positive (Arimatsu, Kaewkes, Laha, Hong, & Sripa, 2012). Also, NGS method was used for *in silico* analysis of >50,000 sequences of *O. viverrini* genome, which can be employed for future diagnostic studies (Young et al., 2010).

At the moment, only an ELISA kit (Vitrotest Anti‐Opisthorchis kit) is commercially available in the market to detect *O. felineus*.

#### 2.1.3 *Paragonimus* spp.

*Paragonimus* spp. is a crustacean‐borne flatworm and a lung fluke that is the causative agent of paragonimiasis in humans (Diaz, 2013). Embryonated eggs of *Paragonimus* spp. are converted to miracidia, which infect snails, the first intermediate host. Then, the cercariae emerge from the snails and infect crustaceans such as crabs or crayfish, the second intermediate host. Finally, humans may become infected as the definitive host after ingesting insufficiently cooked infected crustaceans. The metacercariae subsequently penetrate the duodenum, the diaphragm, and the parietal pleura and mature within the pleural space or the lung. As a result, the parasite eggs are coughed up as bloody sputum or swallowed and later excreted in the feces. The clinical features of paragonimiasis appear between two and 16 weeks post infection and include fever, cough, hemoptysis, and peripheral eosinophilia.

Currently, diagnosis of paragonimiasis is performed by microscopic egg examination in stool or pleural fluid samples (Slesak et al., 2011). The gold standard for the detection of *Paragonimus* spp. is the detection of at least one parasite egg in any sample by three different methods, including wet film (WF), Ziehl‐Neelsen stain (ZNS), and a formalin‐ether concentration technique (FECT). A western blot assay has been designed to detect *P. kellicotti* and *P. westermani* infections for the serologic diagnosis of paragonimiasis (Fischer et al., 2013). This method depends on the identification of the ES Ags of the parasite. Fourteen proteins with a molecular mass of 4 to 62 kDa were detected by serum antibodies from patients with a *P. kellicotti* or *P. westermani* infection. Three polypeptides of 21, 23, and 34 kDa were recognized by all 11 sera from demonstrated *P. kellicotti* cases. In addition, a PCR‐based diagnosis method was designed for the molecular detection of paragonimiasis (Intapan et al., 2012). In this assay, the genomic DNA was isolated from the eggs in the patient sputum obtained during a bronchoscopy. The primers were designed for the ribosomal ITS2 sequence, which is identical in both *P. pseudoheterotremus* and *P. heterotremus*, and a specific region of the *P. pseudoheterotremus cox1* gene. Sequencing of the PCR products showed a 99%–100% and 98%–99% identity for the *ITS2* and *cox1* genes, respectively.

Most recently, a NGS method has been used to identify the transcripts and predict the proteins expressed in adult *Paragonimus* spp. (McNulty et al., 2014). These novel techniques may provide useful tools for diagnostic purposes in the future.

At the moment, only *P. westermani* IgG and IgM ELISA kits are available in the market for diagnosis or research purposes.

### 2.2 Cestoda

#### 2.2.1 *Diphyllobothrium* spp.

Diphyllobothriosis is a zoonotic fish‐borne parasitic infection of fish and mammals caused by fish tapeworms belonging to the genus *Diphyllobothrium* spp., including *D. latum* and *D. nihonkaiense*. The coracidia of *D. latum* hatched from eggs in the water are ingested by crustaceans in water, the first intermediate host (Scholz, Garcia, Kuchta, & Wicht, 2009). The ingested parasites are converted to procercoid larvae in the body cavity of the crustaceans. The infected crustaceans are ingested by freshwater fishes, the second intermediate host, in which the procercoid larvae develop into plerocercoids. The parasite life cycle may be completed in humans, the ultimate host when they consume raw or undercooked fish. The common symptoms of diphyllobothriosis include diarrhea or constipation, abdominal pain, and pernicious anemia due to the excessive consumption of vitamin B12 by the helminth (Waki, Oi, Takahashi, Nakabayashi, & Kitani, 1986).

The routine diagnostic method for diphyllobothriosis is identification of the eggs and proglottid segments in stool samples by direct microscopic examination (Waki et al., 1986). Another direct identification method is radiological imaging using an intraduodenal injection of diatrizoic acid (Hypaque or Gastrografin) for the visualization of the attachment of Diphyllobothrium to the duodenum. Additionally, an evaluation of the blood vitamin B12 level could be another nonspecific method for the diagnosis of diphyllobothriosis.

Because of the morphologic similarities between different Diphyllobothrium tapeworms, species‐specific diagnosis is challenging (Chen et al., 2014). More recent studies have preliminarily identified two Diphyllobothrium specimens by conventional morphological methods, and the identity of species was then confirmed by a molecular approach involving the 18S rDNA partial sequence, complete sequences of ITSs and 5.8S rDNA, partial sequences of mitochondrial cox1, and the mitochondrial NADH dehydrogenase subunit 5 (nad5) (Guo et al., 2012). To date, no reports of serologic or coproantigenic diagnostics of diphyllobothriosis exist.

At present, there is no commercial detection kit in the market for diagnosis of diphyllobothriosis.

#### 2.2.2 *Taenia* spp.

*Taenia* spp. are important tapeworm parasites and the causative agents of zoonotic taeniasis and cysticercosis in humans. The most important zoonotic foodborne species transmitted from animal‐derived foods to humans are *T. asiatica* (an Asian tapeworm)*, T. saginata* (a beef tapeworm), and *T. solium* (a pork tapeworm) (Galan‐Puchades & Fuentes, 2013). The eggs or proglottids of the parasite enter the environment via the feces of infected humans. The eggs or proglottids are transmitted to cattle (*T. saginata*) and pigs (*T. solium, T. asiatica*) as intermediate hosts by vegetation. Oncospheres are hatched from the eggs in the intestinal walls of the cattle and pigs. The oncospheres further develop into cysticerci in the muscles of the intermediate host. Finally, humans (the definitive host) can become infected by ingesting raw or uncooked infected meat. The swallowed larvae attach to the small intestine mucosa by the head (scolex) and form proglottids. At approximately 2 months post infection, the proglottids detach and are excreted in the feces. The symptoms of taeniasis are abdominal pain, diarrhea, distension, and nausea, but sometimes no symptoms occur (Garcia, Gonzalez, Evans, & Gilman, 2003; Garcia, Gonzalez, & Gilman, 2003; Geysen et al., 2007). Humans can also act as an intermediate host for *T. solium*, when cysticerci are formed in organs including the brain.

Visual meat inspection has been the most popular method to control the transmission of Taenia in humans, even though it lacks both accuracy and sensitivity (Galan‐Puchades & Fuentes, 2013). At the least, there is a need to increase the area and number of prediction sites observed during inspection to improve the meat inspection procedure (Wanzala et al., 2003). Direct microscopic (Jeri et al., 2004) and ELISA (Allan, Wilkins, Tsang, & Craig, 2003) assays have been considered as gold standards for the diagnosis of taeniasis in humans. An enzyme‐linked immunoelectrotransfer blot (EITB) was developed using soluble *T. solium* metacestode antigen (Pathak, Allan, Ersfeld, & Craig, 1994) for the serodiagnosis of taeniasis. The respective sensitivity and specificity were 90% and 100%, but were 70% and 73% for the ELISA. Two antigens, HP6‐2 (a major protein) and Ts45S‐10 (an antigen homologous to the 45S antigen of *T. ovis*) were used in a different serodiagnosis method (Abuseir, Kuhne, Schnieder, Klein, & Epe, 2007). A sensitivity of 100% and a 98% specificity for the HP6‐2 ELISA was found for serum samples. Meat juice samples had a slightly lower specificity of 95%. Also, *T. solium* cAMP‐dependent protein kinase regulatory subunit (TsPKA‐r) as an ES antigen was applied for serodiagnosis of porcine cysticercosis (Liu et al., 2017). The designed indirect ELISA showed good sensitivity and specificity (93.88% and 96.40%, respectively) in serodetection of pigs *T. solium* metacestodes, and no cross‐reaction was observed with other Taenia species, such as *T. hydatigena*. In addition, nanobodies as the camelid‐derived single‐domain antibodies were used for serodiagnosis of cysticercosis of *T. solium* (Deckers et al., 2009). Although, the developed sandwich ELISA showed no cross‐reaction with the other species of Taenia including *T. hydatigena, T. saginata,* and *T. crassiceps*, the results revealed that this method needs further validation to improve the sensitivity. Cysts were collected form slaughtered animals for the molecular diagnosis of taeniasis, and DNA was isolated from them (Abuseir, Epe, Schnieder, Klein, & Kuhne, 2006). Two primer sets for HDP‐1 and HDP‐2 were designed for a genomic DNA fragment. As a result, HDP‐1 primers could detect 200 fg of DNA of *T. saginata* and 100 pg of DNA of *Cysticercus bovis* (cysticercus or larval stage of *T. saginata*), whereas HDP‐2 could detect 1 pg of DNA of *T. saginata* and 1 ng of DNA of *C. bovis*. In another PCR‐based diagnosis method, the mitochondrial *cox1* gene was used to design a primer (Chiesa et al., 2010). Using this method, 94.7% of the samples (162/171) showed taenia infection, and it was proposed that the *cox1* gene might be a suitable target for the molecular diagnosis of taeniasis. Moreover, PCR‐RFLP has been considered as another molecular diagnostic method of taeniasis, especially to differentiate between *T. saginata* and *T. solium* proglottids. In this method, PCR was followed by treatment with the restriction enzyme *Ddel* (Rodriguez‐Hidalgo, Geysen, Benitez‐Ortiz, Geerts, & Brandt, 2002). A nested PCR method has also been designed for the detection of a *T. solium* oncosphere‐specific protein, the *Tso31* gene. This method did not cross‐react with *T. saginata* or other parasites (Mayta et al., 2008).

At present, only an ELISA kit is commercially available in the market for diagnosis of produced IgG against cysticerci of *T. solium* in human serum.

### 2.3 Nematoda

#### 2.3.1 *Anisakis simplex*

The nematode *Anisakis simplex* is the causative agent of anisakiasis, which may be contracted from eggs in the water excreted by infected marine mammals. Larvae hatch from the eggs and become free‐swimming. The larvae are ingested by crustaceans (an intermediate host), and the infected crustaceans are eaten by fishes (another intermediate host), in which the larvae migrate to the muscle tissue. The larvae are also transferred to other fishes and squids via predation. The larvae are transmitted to marine mammals and humans (the definitive hosts) via the ingestion of infected fish and develop into adult helminths. The most common symptoms of human anisakiasis are gastrointestinal problems, including eosinophilic intestinal phlegmon, acute abdominal pain, and allergic reactions (Audicana & Kennedy, 2008; Moreno‐Ancillo et al., 1997).

The current method for the diagnosis of anisakiasis is based on gastroscopy and visual inspection of the larvae or a histopathology analysis of a biopsy of infected tissue (Gamboa et al., 2012). The parasite induces the production of immunoglobulin E (IgE) in infected human blood. Determination of the antibody concentration by IgE immunoblotting, a radioimmunoassay (RIA), a radioallergosorbent test (RAST), an enzyme‐linked immunosorbent assay (ELISA), and an immunofluorescent antibody assay (IFA) has been extensively used (Dominguez Ortega et al., 2000; Sakanari et al., 1988). In addition, the production of recombinant allergens by the parasite, such as *Ani s1* and *Ani s7,* and their use in a skin prick test have shown that *Ani s1* and *Ani s7* could, respectively, detect 82.1% and 92.9% of gastro‐allergic anisakiasis cases (Cuellar et al., 2012). In addition, recombinant *Ani s1* and *Ani s7* were used in the production of a highly sensitive ELISA kit for the serologic detection of IgE in human anisakiasis (Cuellar et al., 2012).

Now, at the present moment, there is no commercial detection kit in the market for diagnosis of anisakiasis.

#### 2.3.2 *Gnathostoma* spp.

Parasitic nematodes *Gnathostoma* spp. are the causative agents of gnathostomiasis. Human exposure to this parasite is highly dependent on the ingestion of undercooked meat, including shrimp and fish. Felines and canines are the natural definitive hosts for *G. spinigerum*, and pigs are the definitive host for *G. hispidum*. Additionally, humans act as a definitive host. Unembryonated eggs are transmitted to the water via the feces of infected felines, canines, and pigs. The eggs become embryonated and hatch in the water, and the larvae are released from the eggs. The larvae are ingested by copepods (the first intermediate host) and develop to the next larval stage. After ingestion of the copepods by frogs and fishes (the second intermediate hosts), the L2 larvae developed into L3 larvae. Finally, the infected second intermediate hosts are ingested by the definitive hosts (marine predator birds, canines, felines, and humans), and the infection is transmitted to them. The clinical features of gnathostomiasis include various cutaneous, neurological, ocular, and visceral symptoms (Herman & Chiodini, 2009).

This disease may include eosinophilic meningoencephalitis, the diagnosis for which is based on the identification of eosinophils in the cerebrospinal fluid (CSF) (Graeff‐Teixeira, da Silva, & Yoshimura, 2009; Kuberski, 1979). The eosinophil count should be higher than 10 eosinophils per ml or 10% of the total CSF leukocyte count. A western blot with purified cysticercus glycoprotein antigens of *Gnathostoma* spp. is the gold standard for the diagnosis of gnathostomiasis (Graeff‐Teixeira et al., 2009). An immunoblotting analysis has been designed as the gold standard with 100% sensitivity and specificity for the detection of specific IgG against the antigenic components of *G. spinigerum* (Intapan et al., 2010). An immunoblotting assay detected antigenic bands with molecular masses ranging from 21 to >110‐kDa. The 21‐ and 24‐kDa bands were determined to be polypeptides useful for the diagnosis of neurognathostomiasis. In another immunoblot analysis, specific IgG antibodies were identified against a *G. spinigerum* crude larval extract and a recombinant matrix metalloproteinase (rMMP) protein. The specificity and sensitivity for both antigenic samples were 100%, and it was suggested that the anti‐MMP IgG antibody could be used as an alternative diagnostic method (Janwan et al., 2013). Several other immunological techniques have been designed, such as an ELISA for the detection of IgG antibodies and a two‐dimensional PAGE method for the identification of serum antigens. None are available as commercial kits on the market (Graeff‐Teixeira et al., 2009).

At the moment, there is no commercial detection kit in the market for diagnosis of gnathostomiasis.

#### 2.3.3 *Trichinella* spp.

*Trichinella* spp. are the causative agents of a zoonotic helminthic trichinellosis. Of the main sources of *Trichinella* spp. (including mammals, birds, and reptiles (Alban et al., 2011)), the most important source of human infection worldwide is the domestic pig, whereas horse and wild boar meat have played a significant role during outbreaks in the past three decades in Europe (Gottstein, Pozio, & Nockler, 2009).

The larvae of the parasite are located in the striated muscles (Clausen et al., 1996; Gottstein et al., 2009; Pozio, 2007). Animals and humans become infected after the ingestion of infected meat. The larvae are released from the muscle in the small intestine, where they form adult male and female nematodes that reproduce in the intestinal mucosa. The newborn larvae migrate through the intestinal wall into the lymphatic and blood circulation and finally to the striated muscles, where they form cysts. Trichinellosis is divided into an intestinal and muscular phase (Froscher, Gullotta, Saathoff, & Tackmann, 1988; Gottstein et al., 2009; Kumar, Pozio, de Borchgrave, Mortelmans, & de Meurichy, 1990). The larvae survive in muscle nurse cells for years (up to 40 years in humans and over 20 years, for example, in polar bears). The larvae of *Trichinella* and their metabolites induce the occurrence of some immunological, pathological, and metabolic disorders, such as gastroenteritis (abdominal pain and diarrhea), during the acute phase of the infection (Gottstein et al., 2009), and deaths have even been reported (Dupouy‐Camet, 2002).

Of 12 different genetically detected *Trichinella* spp., *Trichinella spiralis* is the most common species in domestic and wild swine and is also the major etiological agent that causes the broad global distribution of trichinellosis in humans (Pozio & Darwin Murrell, 2006). Trichinellosis can be diagnosed by several methods. In the slaughterhouse, the artificial digestion of meat using a magnetic stirrer combined with microscopy is the preferred diagnostic method (Alban et al., 2011; Van Der Giessen et al., 2013). Although the sensitivity of this detection assay is 40% for less than one larva per 100 grams of infected meat, it is 100% for more than one larva per 100 grams of infected meat. Trichinella can also be diagnosed by several other methods, some of which might offer reasonable options to the current methods for meat inspection. Detection of anti‐*Trichinella* immunoglobulin G (IgG) in animal serum or detection of the parasite in meat juice by other assays such as ELISA (Gamble et al., 2000; Korinkova, Kovarcik, Pavlickova, Svoboda, & Koudela, 2008; Wang et al., 2017), proteomics (Wang, Cui, Hu, Liu, & Wang, 2014), and molecular methods including PCR, real‐time PCR, LAMP (Lin et al., 2013; Tantrawatpan et al., 2012), and reverse line blot hybridization (RLB) (Rombout, Bosch, & van der Giessen, 2001) can show that an animal is infected with Trichinella. Other methods such as multiplex PCR have focused on the detection of novel molecular markers to differentiate *Trichinella* spp. because the conventional methods have limitations that include labor‐intensiveness, the necessity for a high‐level of expertise with direct microscopic diagnosis, and high costs due to labor requirements. Potential molecular markers include *ITS1* and *ITS2* (Lin et al., 2013; Zarlenga, Chute, Martin, & Kapel, 1999), the mitochondrial *cox3* (Van De et al., 2012), and the aminopeptidase (*TsAP*) (Zhang, Wang, Li, & Cui, 2013) genes, the mitochondrial large subunit ribosomal RNA (*rrnL*) (Blaga et al., 2009; Borsuk, Moskwa, Pastusiak, & Cabaj, 2003; Guenther et al., 2008; Li et al., 2011), the mitochondrial small subunit ribosomal RNA (*rrnS*) (Blaga et al., 2009), the DNA sequence of the 5S rRNA intergenic spacer regions (Rombout et al., 2001), and migratory DNA from *Trichinella* larvae (Li, Wang, & Cui, 2010). An effective strategy for the specific detection of trichinellosis is the identification of a genomic or proteomic determinant absent in the host genome or proteome (Duplessis & Moineau, 2001). In a recently designed molecular method for the identification of *Trichinella* spp., the β‐carbonic anhydrase (β‐CA) genome sequence from *T. spiralis* was used for the PCR‐based detection of trichinellosis (Zolfaghari Emameh, Kuuslahti, Nareaho, Sukura, & Parkkila, 2015). This method had no false‐positive reaction with other parasites, including *T. gondii, T. cati,* and *Parascaris equorum*. Additionally, it was suggested that the β‐CA genomic sequence targeted by PCR could be a reliable genus‐specific method for identification of the trichinella infection of meat in slaughterhouses and the food industry.

At present, there are two types of serologic assays for diagnosis of trichinellosis. The human trichinella IgG ELISA and trichinella antigen test kits.

### 2.4 Protozoa

#### 2.4.1 *Sarcocystis* spp.

*Sarcocystis* spp. are parasitic protozoa and the causative agents of zoonotic sarcocystosis in humans. The taxonomy of Sarcocystis is developing with many species in the genus; 189 species have been listed in 1998 (Poulsen & Stensvold, 2014). The life cycle of some species has been fully described with known intermediate and mediate hosts, while knowledge of others is more fragmentary (Fayer, Esposito, & Dubey, 2015). Man is known to be definitive host only for two Sarcocystis species, *S. hominis* and *S. suihominis*, but has also reported to be (accidental) intermediate host for several unidentified Sarcocystis spp. Sarcocystis species can infect a broad variety of animals including domestic mammals (sheep, cattle, horses, pigs, cats, and dogs), wild mammals (deer), and birds (chickens and ducks). In the life cycle of sarcocystosis, a sexual phase occurs in the intestine of the definitive host. The sporocysts and human oocysts of Sarcocystis are spread to the environment via the feces. The sporocysts and oocysts are transmitted to intermediate hosts after the ingestion of contaminated grass. The sporocysts are ruptured, and the sporozoites are released and infect the endothelial cells of blood vessels and undergo a development stage known as schizogony. Merozoites are released from schizonts, transmitted to muscle cells or other organs, and develop into cysts with bradyzoites. Thereafter, the sarcocystosis can be transmitted to the definitive host after the ingestion of raw or insufficiently cooked meat. Humans can act as either an intermediate host with sarcocysts in skeletal muscle and/or other organs like heart, liver, lung, retina, and kidney or as a definitive host when the parasites occur in the intestine (Dubey et al., 2015). The general symptoms of sarcocystosis depend on which role occurs in the human, but can include myositis, fever, abdominal pain, distension, eosinophilia, and watery diarrhea starting at approximately 1 week post infection (Bunyaratvej, Unpunyo, & Pongtippan, 2007; Fayer, 2004).

Visual inspection for identification of macroscopic cysts of *Sarcocystis* spp. in the tongue, esophagus, and hearts of animals is the preferred method for detection of sarcocystosis in animal carcasses (Bittencourt et al., 2016). Some species produce only microscopic cysts that cannot be observed by visual inspection. One study used a direct method for the diagnosis of sarcocystosis based on a biopsy from infected meat and a microscopic examination after H&E and Periodic Acid‐Schiff (PAS) staining. A different direct method for detection of sarcocysts used a pepsin‐digestion assay and had a higher sensitivity than the muscle squash technique (Hamidinejat et al., 2013; Nourollahi Fard, Asghari, & Nouri, 2009). An antigen was isolated from *S. muris* cystozoites for the serologic detection of sarcocystosis and was used in an ELISA and an indirect fluorescent antibody test (IFAT) (Tenter, 1987). Unfortunately, both serologic identification assays cross‐reacted with *T. gondii*. The contradictory results indicated that these serologic techniques cannot be considered as gold standards for the identification of sarcocystosis. A new report suggested a high prevalence of *S. hominis* in the European beef market (More et al., 2014). Meat specimens from different supermarkets and butcheries in Germany were evaluated by microscopic and molecular methods including conventional and multiplex real‐time PCR. The target gene for the PCR diagnosis method was the 18S ribosomal DNA (rDNA). The results showed that 52%, 37%, 6.6%, and 6.2% of the meat samples were positive for *S. cruzi, S. sinensis, S. hirsuta,* and *S. hominis* (Vangeel et al., 2007). Among these species, *S. cruzi, S. hirsuta,* and *S. hominis* mainly use cattle as their intermediate host (Dubey & Lindsay, 2006). In addition, *S. sinensis* was the most prevalent thick‐walled *Sarcocystis* spp. in beef available in German markets. An 18S rRNA sequence was used for primer design in Belgium and a PCR‐based technique for the diagnosis of sarcocystis infection was tested in minced beef (Vangeel et al., 2007). The results showed sarcocystis infection in 97.4% of meat specimens, which was a critical warning for European countries to perform efficient screening in their meat markets for sarcocystosis detection. The goal could be the detection of species infective to humans via animal carcasses. Hence, it is better to understand more about Sarcocystis epidemiology with new molecular tools and discover their actual impact on human health. In addition, a recently released study showed food poisoning in humans via the ingestion of raw horsemeat infected with *S. fayeri* (Kamata et al., 2014). This finding identified a 15‐kDa protein from *S. fayeri* cysts that was homologous to the actin‐depolymerizing factor of *T. gondii* and *Eimeria tenella*.

At present, there is a commercial PCR kit (VetPCR^TM^) for molecular detection of *S. neurona* in equine protozoal myeloencephalitis.

#### 2.4.2 *Toxoplasma gondii*

*Toxoplasma gondii* is an intracellular protozoan and the causative agent of human toxoplasmosis. Parasite oocysts are released in the feces of felines and ingested by other warm‐blooded animals such as birds, pigs, and sheep. Sporozoites are released from the oocysts in the intestine and invade the intestinal epithelial cells where they divide and differentiate into tachyzoites. The tachyzoites develop to bradyzoites and form cysts in the brain, liver, and muscles. Although the ingestion of both tissue cysts with bradyzoites and oocysts can be a source of toxoplasmosis, humans are usually infected after the consumption of infected meat. Fetal abnormalities might result from the infection of a pregnant woman with the parasite, such as spontaneous abortion or neurological disorders (e.g., blindness, mental retardation, or toxoplasmic encephalitis) (Black & Boothroyd, 2000).

Meat inspection in slaughterhouses does not include *T. gondii* (Lhafi, Mitzscherling, & Kuhne, 2004). A sensitive serologic‐ or molecular‐based method is necessary to change that. Diagnosis of toxoplasmosis in humans can be made by various biological, serological, histological, and molecular methods, or by a combination of them (Van De Ven, Melchers, Galama, Camps, & Meuwissen, 1991), which could also be applied to meat inspection. Direct microscopy is too time‐consuming and insensitive to be used for meat inspection (Udonsom, Buddhirongawatr, & Sukthana, 2010). The Sabin‐Feldman dye test has been the gold standard for the detection of anti‐*Toxoplasma* antibodies in a serologic assay. In fact, several serologic methods are available for the detection of *T. gondii*. The detection of the *P30* gene in ready‐to‐eat meat samples has been shown to be a more sensitive method than the direct isolation of the protozoan (Warnekulasuriya, Johnson, & Holliman, 1998). Additionally, *T. gondii* was detected in commercial cured ham samples by a mouse concentration bioassay and an indirect immunofluorescence technique (Bayarri, Gracia, Perez‐Arquillue, Lazaro, & Herrera, 2012). Moreover, a rapid immunochromatographic strip (ICS) has been designed to detect *T. gondii* circulating antigens in animal blood during the acute stage of infection (Wang et al., 2011). The ICS has been compared with an ELISA method. In 381 serum samples, the positive rates of the ICS and ELISA were 5.2% and 5.8%, respectively. Another serologic diagnostic method for *T. gondii* is a rapid one‐step sandwich ELISA, which was designed to detect a soluble antigen (CAg) from infected blood and tissues (Chen et al., 2008). A magnetic‐capture polymerase chain reaction (MC‐PCR) was used to detect *T. gondii* DNA in 231 horse meat samples from French supermarkets for the molecular identification of toxoplasmosis, and the results were compared with an ELISA (Aroussi et al., 2015). The MC‐PCR detected *T. gondii* DNA in 43% of the horse meat samples, while the ELISA was able to detect the infection in 13%–90% of the samples. Therefore, no correlation was identified between the MC‐PCR and ELISA assays for *T. gondii* DNA. In addition, *T. gondii ITS1*‐derived primers and a fluorogenic probe have been used to detect the protozoan infection in mice and pork meat samples by real‐time PCR (Jauregui, Higgins, Zarlenga, Dubey, & Lunney, 2001). This assay was able to detect one *T. gondii* bradyzoite, which is equivalent to 0.1 pg of *T. gondii* genomic DNA. It was noted that toxoplasmosis could be identified by several other methods, such as inoculation in mice, tissue culture, and a hybridization assay, but these are too slow and expensive for routine meat inspection and are not considered as suitable choices for food monitoring (Van De Ven et al., 1991).

Recently, NGS for whole genome of *T. gondii* has been applied to differentiate between virulent Wh3 and less virulent Wh6 strains (Cheng et al., 2015). It is conceivable that these novel techniques will offer excellent choices for diagnostic purposes in the future.

At present, there are different types of commercial rapid, serologic, and molecular methods for detection of *T. gondii* in the market: TORCH rapid test, latex agglutination test (Pastorex™) for detection of infection in serum, ELISA assay for detection of IgG antibodies, and PCR method.

## 3 CONCLUSIONS

The “top ten” foodborne parasites of the FAO‐WHO in July 2014 (FAO, 2014) indicate that parasites including *T. solium, T. gondii, T. spiralis,* and *O. viverrini* are the most important to be diagnosed in animal‐derived foods.

The diagnostics of foodborne parasites in meat inspection mainly relay on conventional, visual, and direct microscopic methods. A meat inspection decision is an official authoritative order based on existing legislation, and new methodology must be carefully evaluated first and accepted by legislation. Today, direct visual methods are inevitable meat inspection processes for a single carcass in slaughterhouses. However, new diagnostic methodologies will afford more tools for this in the future.

Promising results have been obtained with high sensitivity and specificity and are the main reasons that the FAO, the WHO, and the OIE (World Organization for Animal Health) have published new guidelines for the surveillance, management, prevention, and control of many parasitic infections, including, taeniosis (Gilman, Gonzalez, Llanos‐Zavalaga, Tsang, & Garcia, 2012), trichinellosis (Gottstein et al., 2009), echinococcosis (Van Kesteren et al., 2015), and trypanosomiasis (WORLD HEALTH, 2013), and molecular methods have been approved as valid identification tools for the identification of parasite species.

These methods include commercial serologic detection kits and DNA‐based molecular research techniques, which should be automated for large‐scale use in food production from animal origins as well as research food and quality control laboratories of food production companies.

Additionally, these methods can be applied now to develop selected parasite‐free farming systems for specific consumer groups, although a study of farming conditions revealed that their current status does not guarantee the production of *Toxoplasma*‐free pork (Djokic et al., 2016).

Moreover, to find the best surveillance system for production and consumers, more information about the molecular biology and epidemiology of parasites is required, along with a better understanding of the impact of parasites on human health.

## CONFLICT OF INTEREST

The authors declare that they have no conflict of interests.
